# Olfactory, Auditory, and Vestibular Performance: Multisensory Impairment Is Significantly Associated With Incident Cognitive Impairment

**DOI:** 10.3389/fneur.2022.910062

**Published:** 2022-07-11

**Authors:** Jacob C. Lucas, Zack Arambula, Alexandra M. Arambula, Katherine Yu, Nathan Farrokhian, Linda D'Silva, Hinrich Staecker, Jennifer A. Villwock

**Affiliations:** ^1^Department of Otolaryngology- Head and Neck Surgery, University of Kansas School of Medicine, Kansas City, MO, United States; ^2^Department of Physical Therapy and Rehabilitation Science, University of Kansas Medical Center, Kansas City, MO, United States

**Keywords:** multisensory impairment, age-related hearing impairment, vestibular impairment, olfactory impairment, cognitive impairment, AROMA, Montreal Cognitive Assessment (MoCA)

## Abstract

**Background:**

Dysfunction in the olfactory, auditory, and vestibular systems are commonly seen in aging and are associated with dementia. The impact of sensory loss(es) on cognition is not well understood. Our aim was to assess the relationships between performance on objective multisensory testing and quantify the impact of dysfunction on cognition.

**Methods:**

Patients presenting with subjective hearing loss presenting to a tertiary care otologic/audiologic clinic were identified and underwent multisensory testing using the Affordable, Rapid Olfactory Measurement Array (AROMA), pure tone audiometric evaluations, and the Timed “Up and Go” test. Cognitive impairment (CI) was assessed *via* the Montreal Cognitive Assessment (MoCA) was also administered.

**Key Results:**

180 patients were enrolled. Thirty one percentage (*n* = 57) screened positive for cognitive impairment. When evaluating single sensory impairments, we found that olfactory dysfunction, gait impairment, and sensorineural hearing loss were all statistically significantly (*p* < 0.05) associated with a higher risk of cognitive impairment (ORs 3.89, 3.49, and 2.78, respectively) for CI. Multisensory impairment was significantly associated with cognitive impairment. Subjects with dysfunction in all domains were at the highest risk for cognitive impairment (OR 15.7, *p* < 0.001) vs. those with impairment in 2 domains (OR 5.32, *p* < 0.001).

**Conclusion:**

Dysfunction of the olfactory, auditory, and vestibular systems is associated with a significantly increased risk of CI. The dramatically increased risk of CI with multisensory dysfunction in all three systems indicated that MSD may synergistically contribute to CI.

## Introduction

Cognitive impairment (CI) and dementia have been linked to sensory impairments in multiple domains such as hearing loss, olfactory dysfunction, and vestibular dysfunction. Several large, epidemiologic studies suggest that impairments in more than one sensory domain may be additive with respect to subsequent risk of developing CI. Unlike other risk factors such as age and genetics, sensory deficits are potentially modifiable by techniques such as hearing amplification, olfactory training, and vestibular therapy. Emerging literature suggests that approaches to improve sensory processing, such as cochlear implantation, may reverse cognitive decline (1).

The concept of Multisensory Impairment has been broached in the literature and is an area of active study; several retrospective cohort studies have examined age-related sensory decline including the Epidemiology of Hearing Loss Study (EHLS) (2, 3), Health Aging and Body Composition (Health ABC) (4), National Social Life, Health and Aging Project (NSHAP) (5), and Baltimore Longitudinal Study of Aging (6). Dual impairments in hearing and vision have even been associated with increased risk of all-cause mortality (7). The additive effects of multiple sensory impairments is compounded when compared to loss of a single sense (2). Sensory domains examined include hearing, touch, taste, olfaction, and vision (4, 5, 7, 8). Balance, gait, and vestibular function are also implicated and associated with age-related cognitive decline (6, 8–10). In the context of multisensory impairment, no studies have examined the summative effects of hearing, balance and gait, and olfaction on incident cognitive impairment.

The objective of this study was to prospectively examine neurocognition, olfactory performance, and gait and balance in subjects presenting for audiologic evaluation to determine the incidence of multisensory impairment in this population and the correlation of sensory impairments with neurocognitive status.

## Methods

Institutional review board approval was obtained prior to commencement of any study activities (IRB #145682). This was a cross-sectional case-control study with initial recruitment occurring over a 6-month period from February 2021 through June 2021.

### Subjects

Subjects were recruited from a patient pool presenting with chief complaint of “Hearing Loss” to an otology and audiology clinic. Subjects presenting for audiologic evaluation were screened for eligibility and provided with written informed consent to participate. Enrolled subjects underwent evaluation of hearing, gait and balance, olfaction, and cognitive function. Exclusion criteria were age <50, history of primary progressive neurological disease such as Parkinson's and Multiple Sclerosis, non-ambulatory patients, patients that were unable to follow instructions due to severe cognitive impairment, conductive hearing loss due to middle ear pathology, non-intact tympanic membrane, vestibular schwannoma or other central nervous system tumor, and recent COVID-19 infection. Hearing was assessed using a comprehensive audiologic evaluation including audiometric thresholds, pure tone average, speech discrimination, and tympanometry. Olfaction was assessed using the Affordable, Rapid, Olfactory Measurement Array (AROMA) test (11, 12). Gait and balance were assessed with the Timed Up-and Go assessment (13). Cognitive function was evaluated using the Montreal Cognitive Assessment (MoCA) (14). Baseline demographic data was also obtained.

### Audiometric Data

Audiometry was performed in accordance with the American National Standards Institute ANSI/ASA S3.21-2004 (R 2019) standards (15) and the American Speech-Language-Hearing Association guidelines (16). Hearing level (HL) thresholds measured in decibels (dB) were obtained at 250, 500, 1,000, 2,000, 3,000, 4,000, 6,000, and 8,000 Hertz (Hz). Air and bone conduction thresholds were measured in both ears. Pure-tone averages (PTA) were calculated for each individually tested ear using 500, 1,000, 2,000, and 3,000 Hz, as recommended by the American Academy of Otolaryngology–Head and Neck Surgery (AAO-HNS) (17) and the American Medical Association (18). If no recording existed of the 3000 Hz threshold, the mean of the 2,000 and 4,000 Hz thresholds was utilized; a PTA valuation using this average is within +/- 5 dB of the PTA utilizing 3,000 Hz in 99% of audiograms in one previous series of 2170 patients (19). Word recognition scores were also obtained. A PTA > 25 dB HL was considered to have a sensorineural hearing impairment. For purposes of analysis, the best hearing ear was used for subject classification.

### Timed Up-and-Go

The Timed “Up & Go” (13) test was chosen as a simple screening tool for balance impairment due to its ease of administration and well-established cut off scores for patients with vestibular dysfunction. It is a timed version of the older “Get Up and Go” test (20). The TUG consists of the average time of three trials: the patient is instructed to sit in a chair, a timer is started, the patient stands, walks 3 meters, then turns around and walks back to the chair, and finally sits back down (13). The TUG is well-studied and has excellent validity and reliability in a wide range of adult populations with various disabilities, including Parkinson's, cerebral palsy, and stroke. It has also been studied in otherwise “normal” elderly adult populations (21). It had a sensitivity of 80% in determining fall risk for patients with vestibular disorders, making it a reasonable choice for balance screening. Fall risk due to impairment was associated with a TUG score > 11 s (22). For this reason, subjects with TUG scores > 11 s were considered to have gait and balance impairment during analysis.

### Affordable, Rapid, Olfactory Measurement Arrays Testing

AROMA (11) is an essential oil-based test that comprises 14 scents and one negative control at four concentrations: 1X, 2X, 4X, and 8X. It is administered by trained research personnel. The full battery consists of four rounds of 15 inhalant sticks. The number of odorants and concentrations included in olfactory testing is dependent on each subject's baseline olfactory status; participants start at the 2X concentration and are presented all scents in randomized order. Patients select answers from a 4-item multiple-choice field. Incorrect responses trigger a higher concentration to be presented in the next round. Correct responses trigger the lower concentration to be presented. Due to this branching logic and the nested quality of the testing array, every test is unique, and requires administration on a tablet computer. All participants are asked to identify the 2X concentrations, but subsequent rounds are customized based on responses. This allows increased stratification of responses and development olfactory phenotypes. Preliminary data has identified unique testing phenotypes for normal, mild cognitive impairment, and Alzheimer's disease patients (12). Out of a total score of 100, patients were classified as normosmic (>75), hyposmic (< 75), and anosmic (<40). During multivariate modeling, anosmic and hyposmic patients were collapsed into a single category (Hyposmia) to allow for more straightforward interpretation of binary logistic regression. Related to cognitively impaired individuals, AROMA was validated in examining the relationship of olfactory dysfunction to Alzheimer's dementia, mild cognitive impairment, and cognitively unimpaired individuals. Other advantages of the AROMA are its re-usability, odorant levels that are dynamic, and ability to test both detection and identification of smell (11, 12).

### Montreal Cognitive Assessment

The MoCA, as compared to the Mini Mental Status Exam (MMSE) is more sensitive in the detection of mild cognitive impairment (MCI) (23, 24). It is a multiple domain instrument that tests short term memory recall, working memory, visuospatial ability, abstraction, attention, concentration, and executive functioning (14). It has up to 90% sensitivity in MCI detection and takes ~10 min to administer, making it an ideal cognitive function screening test in the clinical setting. A score of 26 was used as the cutoff for cognitive impairment; (14) subjects with scores <26 were considered CI. The overall score was corrected based on education level–one additional point was added for patients who did not complete high school.

### Data Analysis

#### Ideal Sample Size and Power Analysis

Using previous literature that has estimated the prevalence of various sensory impairments among an aging population, an approximately 30% prevalence of olfactory dysfunction was expected in the control group. Prior prospective cohort studies (2, 3) have demonstrated a Hazard Ratio (HR) of 1.5 for hearing loss and incident dementia. The Odds Ratio (OR) for hearing loss in Alzheimer's dementia was found to be 2.0 in a case-control study with similar design considerations to the present study (25). Risk of fall in a 12-month period for adults aged 65 and older was calculated at 28.7% (26, 27). For the present case-control study comparing multisensory impairment and odds of incident CI; we estimated a likely 0.3 proportion of CI in the control group (28), a 2.0 OR for multisensory impairment and CI, 0.95 confidence level (alpha = 0.05), and 0.8 power level. With these estimates, we determined a conservative sample size of *n* = 275 to uncover a moderate effect size. Patients were actively recruited from a clinical pool of patients presenting with hearing loss. The first author (JCL) screened scheduled clinic lists the day before enrollment for eligible subjects based on chart review. Some initially eligible subjects were ineligible after additional in-person screening. Some subjects declined participation. Some were unable to complete the entirety of testing and so were excluded from analysis. Of the theoretical 585 eligible subjects on preliminary screening during the enrollment period, 180 were included. Enrollment rate based on initial eligibility screening was ~31% and fell short of goal enrollment of 275 subjects.

#### Statistical Analysis

Data was cleaned and wrangled using R (29), RStudio, and the Tidyverse (30) suite of packages. Statistical analysis was performed using base R generalized linear model functions. Plots were generated using the R ggplot2 (31) and audiometry (32) packages.

Wilcoxon Rank Sum, Pearson's Chi-squared, and Fisher's exact test were used where appropriate to evaluate sociodemographic differences between patients with cognitive impairment (Table 1). Univariate analysis was performed for all predictor variables with cognitive status as the outcome variable (Table 2), again utilizing Wilcoxon Rank Sum and Chi-squared testing where appropriate.

**Table 1:** Demography of recruited subjects.

**Characteristic**	**Normal, *N* = 123^a^**	**CI, *N* = 57^a^**	***p*** **-value^b^**
Age at Enrollment	66 (60, 72)	69 (63, 75)	0.064
**Age category**			0.3
50–65	50 (41%)	19 (33%)	
65+	73 (59%)	38 (67%)	
**Sex**			0.7
Female	67 (54%)	29 (51%)	
Male	56 (46%)	28 (49%)	
Black	8 (6.5%)	8 (14%)	0.10
Asian	2 (1.6%)	0 (0%)	>0.9
Native American	1 (0.8%)	0 (0%)	>0.9
White	111 (90%)	49 (86%)	0.4
Other	1 (0.8%)	1 (1.8%)	0.5
**Education level**			**<0.001**
Completed college	47 (38%)	18 (32%)	
Completed high school	34 (28%)	31 (54%)	
Completed graduate/professional degree	39 (32%)	6 (11%)	
Did not complete high school	3 (2.4%)	2 (3.5%)	
**Employment status**			0.12
Retired	68 (55%)	33 (58%)	
Work <40 h per week	15 (12%)	5 (8.8%)	
Work 40 or more h per week	37 (30%)	13 (23%)	
Unemployed	3 (2.4%)	6 (11%)	

a *Median (IQR); n (%)*.

b *Wilcoxon rank sum test; Pearson's Chi-squared test; Fisher's exact test. The bold values indicate the significant p values*.

**Table 2:** Univariate analysis for all measured sensory impairments, with continuous and categorical variables analyzed with Wilcoxan rank-sum and Pearson's Chi-squared, respectively.

**Characteristic**	**Normal, *N* = 123^a^**	**CI, N = 57^a^**	***p*** **-value^b^**
**MoCA score**	28.0 (27.0, 29.0)	24.0 (21.0, 25.0)	**<0.001**
**Timed Up-and-Go**	9.1 (8.1, 10.2)	10.4 (8.8, 13.3)	**0.003**
**PTA (500, 1k, 2k, 3k Hz)**	28 (21, 38)	38 (26, 46)	**0.002**
**Gait status**			**<0.001**
Normal gait	103 (84%)	31 (54%)	
Gait impairment	20 (16%)	26 (46%)	
**Hearing status**			**<0.001**
Normal hearing	58 (47%)	12 (21%)	
SNHL	65 (53%)	45 (79%)	
**Hearing severity**			**0.002**
Normal	58 (47%)	12 (21%)	
Mild	38 (31%)	22 (39%)	
Moderate-severe	27 (22%)	23 (40%)	
**Olfactory status**			**<0.001**
Normal olfaction	58 (47%)	8 (14%)	
Hyposmia	65 (53%)	49 (86%)	
Number of sensory			**<0.001**
**Impairments**			
0 or 1	75 (61%)	11 (19%)	
2	39 (32%)	27 (47%)	
3	9 (7.3%)	19 (33%)	

a *Median (IQR); n (%).*

b *Wilcoxon rank sum test; Pearson's Chi-squared test. The bold values indicate the significant p values*.

Binary logistic regression was chosen for multivariate analysis. Results of multisensory testing, age, gender, and education levels were submitted to binary logistic regression analysis using a stepwise selection procedure. Only items that were related (*p* < 0.10) to the outcome after adjusting for all other items were retained. Odds ratios (OR) for incident cognitive impairment with confidence intervals were calculated for each of two models. Predictor values included hearing impairment, balance impairment, and olfactory impairment, along with control variables for age and education. Each predictor variable was categorized as a binary value and the response variable (cognitive impairment) was also coded as binary, with cases having a MoCA score <26 and controls having scores >26. A first model comparing the outcome for each of three sensory deficits was created, demonstrated in Table 3; a second model comparing the outcome for patients with multiple sensory deficits sought to define the influence of multiple sensory impairments on incident CI demonstrated in Table 4.

Table 3Binary logistic regression model of all sensory impairments, age, and education level; **(A)** Odds Ratios (OR), 95% Confidence Intervals (CI), and p-values for each predictor variable. **(B)** Model coefficients with deviance values for the tested model.

**Table:** 

**A**	**Model 1–binary logistic regression by sensory impairment**		
**Characteristic**	**OR^a^**	**95% CI^a^**	***p*** **-value**
**Hearing loss**			
Normal hearing	—	—	
SNHL	2.78	1.25, 6.52	**0.014**
**Gait and Balance**			
Normal gait	—	—	
Gait impairment	3.49	1.57, 8.00	**0.002**
**Olfaction**			
Normal olfaction	—	—	
Hyposmia	3.89	1.67, 9.94	**0.003**
**Age category**			
50–65	—	—	
65+	0.98	0.45, 2.15	>0.9
**Holds graduate degree**			
No graduate degree	—	—	
Graduate degree	0.22	0.07, 0.58	**0.004**

**Table:** 

**B**	**Model 1–coefficients**.				
	**Estimate**	**Standard error**	**z value**	***p-*** **value**	**Signif**.
(Intercept)	−2.485	0.520	−4.775	0.0000	^***^
Hearing loss	1.023	0.418	2.446	0.0144	^*^
Gait and balance	1.251	0.413	3.029	0.0025	^**^
Olfaction	1.357	0.451	3.012	0.0026	^**^
Age category	−0.021	0.399	−0.053	0.9575	
Holds graduate degree	−1.495	0.515	−2.904	0.0037	^**^

a OR, Odds Ratio; CI, Confidence Interval. Odds Ratios (OR), 95% Confidence Intervals (CI), and p-values for each predictor variable. Signif. codes: 0 <= “***” < 0.001 < “**” < 0.01 < “*” < 0.05 < “.” < 0.1 < “” < 1. Null deviance: 224.8 on 179 degrees of freedom. Residual deviance: 177.6 on 174 degrees of freedom. The bold values indicate the significant p values.

Table 4Binary logistic regression model of *number* of sensory impairments, age, and education level; **(A)** Odds Ratios (OR), 95% Confidence Intervals (CI), and p-values for each predictor variable. **(B)** Model coefficients with deviance values for the tested model.

**Table:** 

**A**	**Model 2–binary logistic regression by number of sensory impairments**		
**Characteristic**	**OR^a^**	**95% CI^a^**	***p*** **-value**
**Number sensory impairments**			
0 or 1	—	—	
2	5.32	2.36, 12.8	**<0.001**
3	15.7	5.54, 48.8	**<0.001**
**Age category**			
50–65	—	—	
65+	0.92	0.42, 1.99	0.8
**Holds graduate degree**			
No graduate degree	—	—	
Graduate degree	0.22	0.07, 0.57	**0.003**

**Table:** 

**B**	**Model 2–coefficients**				
	**Estimate**	**Standard error**	**z value**	***p*** **-value**	**Signif**.
(Intercept)	−1.631	0.376	−4.336	0.0000	^***^
2 Sensory impairments	1.672	0.429	3.898	0.0001	^***^
3 Sensory impairments	2.753	0.551	4.994	0.0000	^***^
Age category	−0.086	0.394	−0.219	0.8264	
Holds graduate degree	−1.511	0.511	−2.959	0.0031	^**^

a OR, Odds Ratio; CI, Confidence Interval. Signif. codes: 0 < = “***” < 0.001 < “**” < 0.01 < “*” < 0.05 < “.” < 0.1 < “” < 1. Null deviance: 224.8 on 179 degrees of freedom. Residual deviance: 179.5 on 175 degrees of freedom. The bold values indicate the significant p values.

In demographic analysis, education level was found to have significant predictive value toward incident cognitive impairment. Additional stratification revealed significant differences between subjects with graduate-level education, and subjects without graduate-level education. Due to this finding, education was added as a control variable to the logistic regression model, with Odds Ratios reported separately. Age was also included as a control predictor variable, with patients stratified into either “50–65” and “65+” age categories.

## Results

One hundred and eighty subjects were enrolled after excluding 4 subjects with incomplete datasets. Fourteen subjects required an assistive device such as a cane or walker to perform the TUG examination. Subject demographic data are presented in Table 1. One hundred and twenty three subjects with normal cognition as defined on the MoCA were enrolled, and 57 subjects with CI were enrolled. The mean age at enrollment was 66 for normal subjects and 69 for CI subjects. Fifty four percentage of subjects were female with 46% male. Recruited subjects were predominantly Caucasian (89%). Education level and employment status were collected during enrollment. Education was found to be significantly associated with differences in incident cognitive impairment, as indicated by Fisher's exact test (two-tailed *p* < 0.001. Other sociodemographic data, including age, gender, race, and employment status were not associated with increased incidence of cognitive impairment.

**Figure 1:**
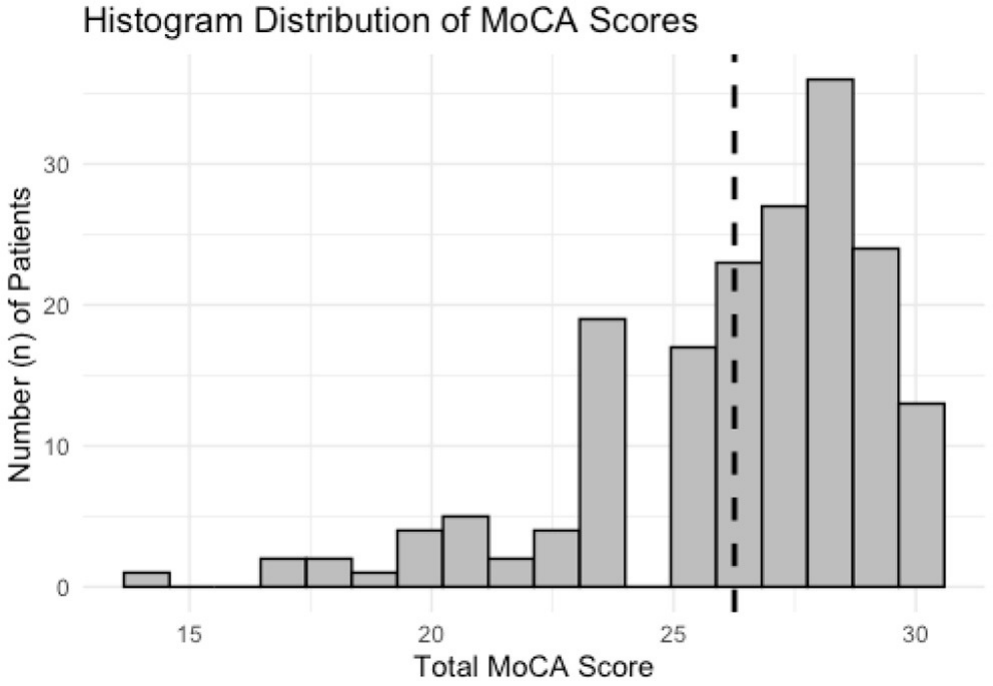
Histogram distribution of MoCA scores for all subjects enrolled for analysis. The data is left-skewed with a heavier distribution of normal and near-normal scores. Dashed line corresponds to a score of 26, scores below which are considered for analysis to have “Cognitive Impairment.”

Total MoCA scores from all subjects are demonstrated in Figure 1. Scores are ordinal and range from 0 to 30. The data is left-skewed with normal or near-normal scores heavily weighted. Cognitive impairment is defined as any score <26. Univariate analysis for all predictor variables on incident cognitive impairment is summarized in Table 2. All three sensory impairments demonstrated significant associations with incident cognitive impairment.

### Hearing

**Figure 2:**
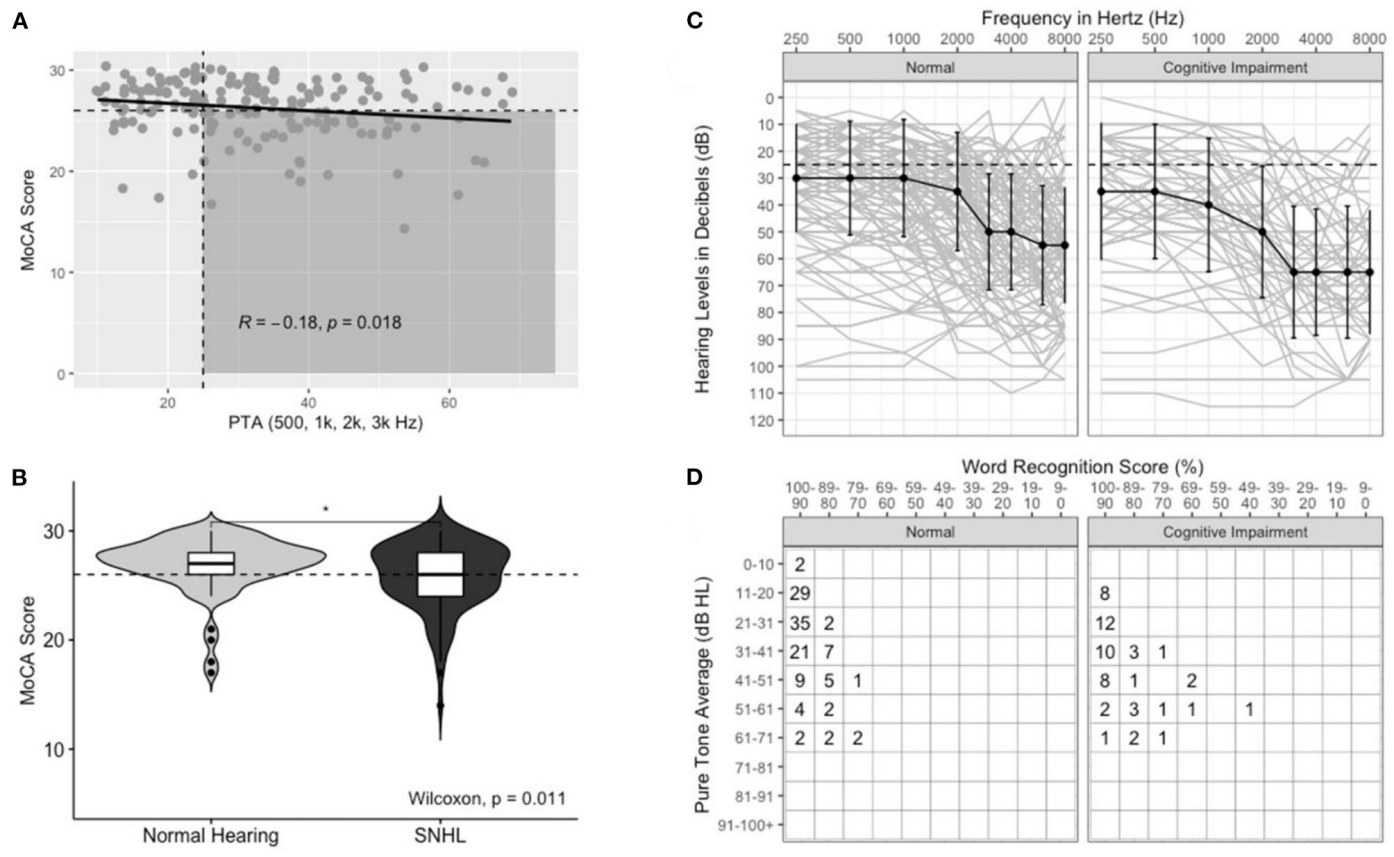
Aggregate audiometric data for patients with Normal Cognition and Cognitive Impairment (CI). **(A)** Scatterplot of PTA versus MoCA score. Horizontal dashed line correlates to MoCA score of 26. Vertical dashed line correlates to PTA of 25. Shaded area includes patients with co-incident hearing loss and CI, line of best fit included with R- and *p*-values for Pearson's correlation. **(B)** Box- and violin-plots of Normal Hearing and Hearing-Impaired (SNHL) individuals and the distribution of MoCA scores in each group. **(C)** Composite audiometric data, median scores for each threshold correspond to the dark line, error bars correspond to 1 SD. **(D)** AAO-HNS minimum reporting standards for raw data of PTA plotted against word recognition scores (WRS) (17).

Hearing-impaired subjects had higher proportions of CI, χ^*2*^(1, *N* = 180) = 11.2, *p* < 0.001. Figure 2 shows aggregate audiometric data for all subjects, with a negative correlation noted between MoCA score and PTA (*R* = −0.18, *p* = 0.018); subjects with impaired hearing on audiometry were more likely to score lower on the MoCA (*p* = 0.011). Composite audiograms for both “Normal” and “CI” subjects are displayed in Figure 2C. Figure 2D displays the minimum standard for reporting hearing loss as prescribed by the AAO-HNS (17). Hearing severity was also significantly associated with cognition, although severity categories were collapsed across moderate, moderately severe, and severe hearing due to a scarcity of subjects with hearing worse than the “Mild” classification. “Normal” and “Mild” hearing loss were heavily weighted in the dataset. No patients with profound hearing loss in the better hearing ear were recruited.

### Gait and Balance

**Figure 3:**
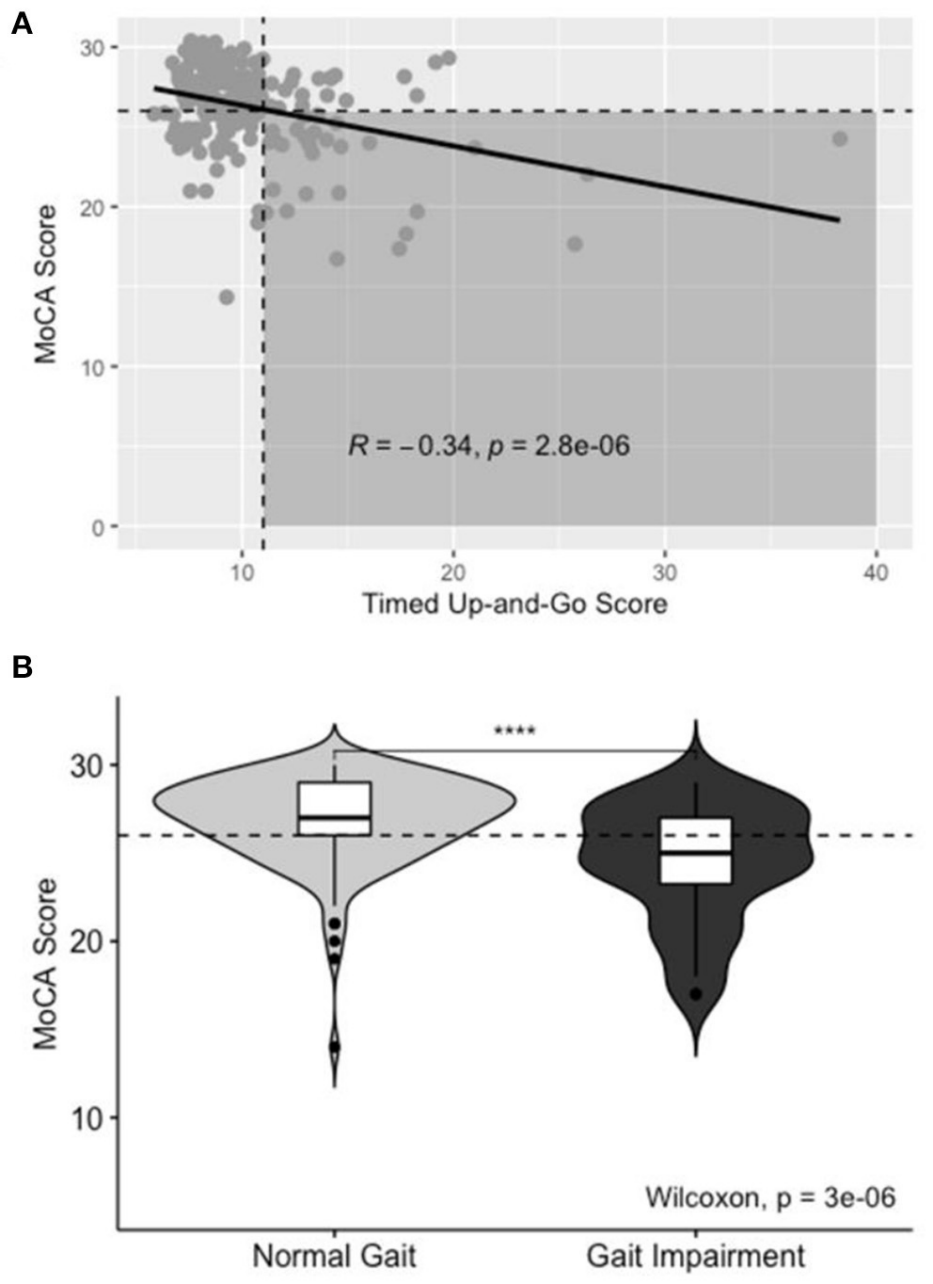
Aggregate TUG data for Normal and Gait-impaired individuals. **(A)** Scatterplot of TUG score vs. MoCA score. Horizontal dashed line correlates to MoCA score of 26. Vertical dashed line correlates to TUG of 11. Shaded area includes patients with co-incident gait impairment and CI, line of best fit included with R- and *p*-values for Pearson's correlation. **(B)** Box- and violin-plots of Normal and Gait-Impaired individuals and the distribution of MoCA scores in each group.

A higher proportion of gait-impaired subjects had co-incident CI on Pearson's Chi Squared test, χ^2^(1, *N* = 180) = 17.6, *p* < 0.001. Gait impairment is depicted in Figure 3; impaired gait scores were associated with lower scores on the MoCA assessment (*R* = −0.34, *p* < 0.001).

### Olfaction

**Figure 4:**
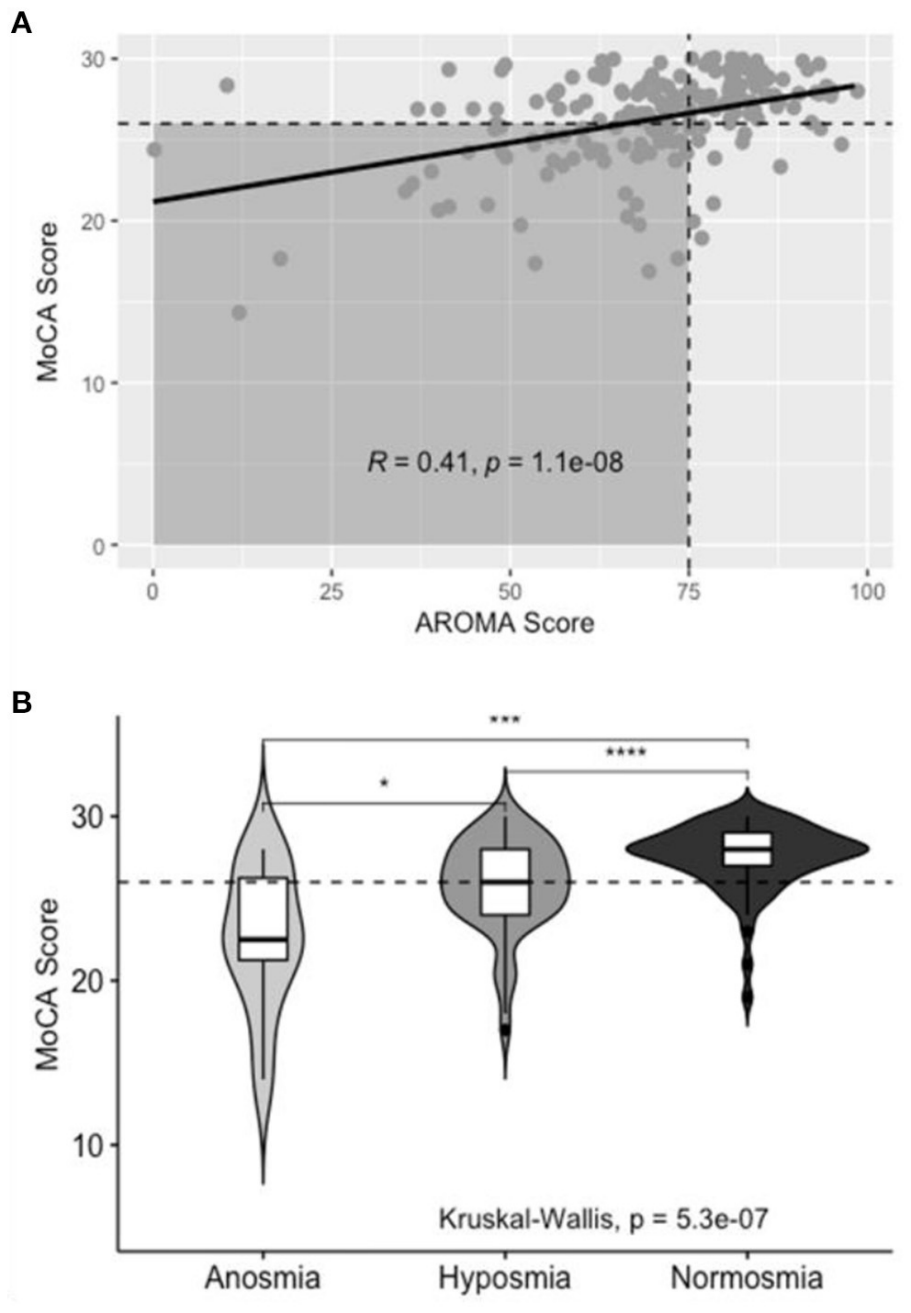
Aggregate AROMA data for subjects with normal and impaired olfaction. **(A)** Scatterplot of AROMA vs. MoCA score. Horizontal dashed line correlates to MoCA score of 26. Vertical dashed line correlates to AROMA score of 75. Shaded area includes patients with co-incident olfactory impairment and CI, line of best fit included with R- and *p*-values for Pearson's correlation. **(B)** Box- and violin-plots of Normal, Hyposmic, and Anosmic individuals and the distribution of MoCA scores in each group.

Olfactory impairment was similarly associated with co-incident CI on Pearson's Chi Squared test, χ^2^(1, *N* = 180) = 18.4, *p* < 0.001. Figure 4 shows the positive correlation between MoCA score and AROMA score (*R* = 0.41, *p* < 0.001). On univariate analysis of olfaction, distinct differences in MoCA score were noted between normosmic, hyposmic, and anosmic individuals; with anosmic individuals scoring lower than hyposmic individuals on the MoCA instrument (Figure 4B). For multivariate modeling using binary logistic regression, anosmic and hyposmic individuals were collapsed into a single “Hyposmia” group to simplify interpretation in the context of other confounding variables.

### Multivariate Modeling–Binary Logistic Regression

Analysis using binary logistic regression is shown in Tables 3, 4. Within initial analysis of sociodemographic data, education level was found to have a significant correlation to cognitive status. Further breakdown demonstrated the stratification to be primarily between individuals with and without graduate-level education. Due to this, education was included as a control variable in the models.

The multivariate influence of three sensory domains on incident cognitive impairment was examined in two separate models.

#### Model 1: Influence of Individual Sensory Impairments

**Figure 5:**
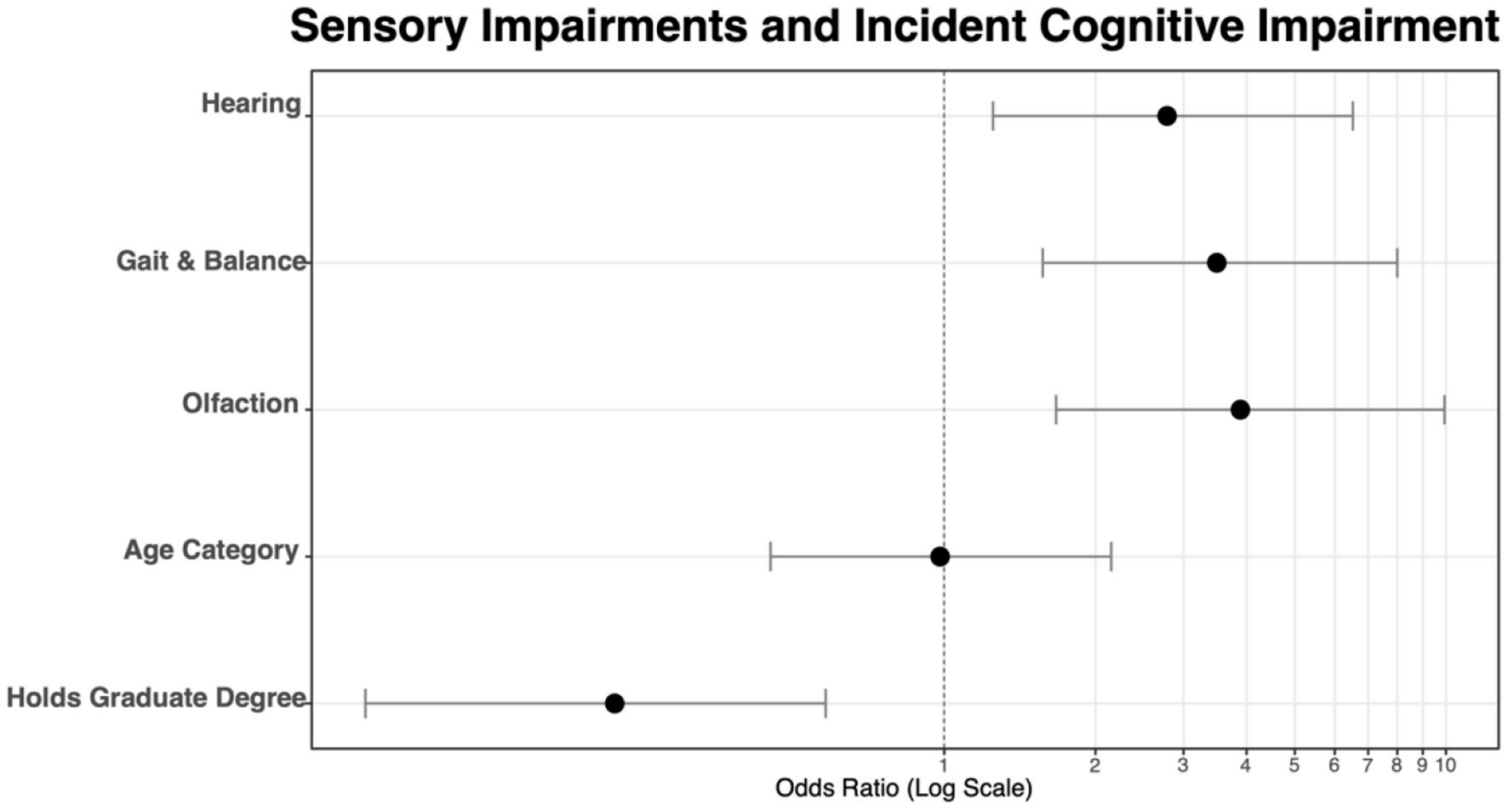
Odds ratios visualized for each of three sensory components and two control variables, age, and education level.

In the first model (Table 3A), the influence of each individual sensory impairment was examined on co-incident cognitive impairment. Hearing loss [OR = 2.78, 95% CI (1.25, 6.52), *p* = 0.014], gait and balance [OR = 3.49, 95% CI (1.57, 8.00), *p* = 0.002], and olfaction [OR = 3.89, 95% CI (1.67, 9.94), *p* = 0.003] were all significant predictors of co-incident cognitive impairment. Having a graduate-level education was associated with a lower odds of co-incident CI [OR = 0.22, 95% CI (0.07, 0.58), *p* = 0.004], while no statistical difference was seen between age groups. Table 3B reports coefficients for the model. Figure 5 depicts the OR values graphically.

#### Model 2: Summative Influence of Multiple Sensory Impairments

The second model sought to examine the additive effects of multiple sensory impairments on co-incident cognitive impairment. To account for the small number of subjects with zero sensory impairments and incident CI (*n* = 2), 0 and 1 sensory impairment categories were collapsed into a single level “0 or 1 Sensory Impairments.” Education and age were again included as control variables in the model. Table 4A displays odds ratios for increasing number of sensory impairments as predictors for co-incident CI. “0 or 1” sensory impairments were considered as the baseline condition. With 2 coexisting sensory impairments, the odds of CI increased [OR = 5.32, 95% CI (2.36, 12.8), *p* < 0.001]. When all 3 sensory impairments were present, the odds increased further [OR = 15.7, 95% CI (5.54, 48.8), *p* < 0.001]. Table 4B reports coefficients for the model.

## Discussion

Despite the well-established link between sensory deficits–many of which are potentially modifiable with training and targeted rehabilitation–and neurocognitive decline, the incidence and impact of multisensory impairments are not well understood. Sensorineural hearing loss increases the relative risk for dementia and has a weighted population attributable fraction of 8.2% among midlife (age 45–65) adults according to the recent Lancet Commission on Dementia Prevention (33), making it the number one modifiable risk factor in the prevention of dementia. Apart from hearing loss, there are no current guidelines related to the screening and treatment of sensory impairments in the context of cognitive decline. The objective of this study was to determine the incidence of hearing, olfactory, and/or balance impairment and determine the magnitude of the impact of single vs. multisensory dysfunction on cognitive performance. When evaluating single sensory impairments, we found that olfactory dysfunction, gait impairment, and sensorineural hearing loss were all statistically significantly associated with a higher incidence of cognitive impairment (OR 3.89, *p* = 0.003; 3.49, *p* = 0.002; and 2.78, *p* = 0.014, respectively) for cognitive impairment. Subjects with dysfunction in all domains were at the highest risk for cognitive impairment (OR 15.7, *p* < 0.001) vs. those with impairment in 2 domains (OR 5.32, *p* < 0.001). These findings underscore the need to comprehensively evaluate patients for multisensory impairment, particularly those who are at-risk for cognitive decline due to advancing age or other known risk factors. Additionally, the sensory domains studied are all amenable to rehabilitation. The dramatically increased risk of cognitive impairment in those with multisensory impairment highlights the potential of sensory rehabilitation, even if only in a single domain, to potentially improve outcomes.

The concept of multisensory impairment is a burgeoning area of active study. For example, several cohort studies have examined age-related sensory decline including the Epidemiology of Hearing Loss Study (EHLS), Beaver Dam Offspring Study (BOSS), Health Aging and Body Composition (Health ABC), National Social Life, Health and Aging Project (NSHAP), and Baltimore Longitudinal Study of Aging (2–6).

### The Correlation Between Sensory Impairments and Cognitive Impairment

There are competing and parallel hypotheses for why sensory impairment contributes to the onset and trajectory of cognitive decline. Most of these use hearing loss as an illustrative example. The “cognitive load” theory postulates that loss of hearing places additional cognitive processing demands on the brain, resulting in diverting of limited neuroprocessing resources toward auditory processing, and away from other cognitive processes such as working memory (34, 35). The cumulative strain may contribute to loss of cognitive function over time. Social isolation due to hearing loss may also contribute to cognitive decline. Self-reported measures such as depression and “fair or poor” overall mental health are seen with a higher frequency in the hearing impaired (36). A common pathophysiologic mechanism underpinning both hearing loss and dementia such as microvascular insult could explain the apparent linkage as well. However, multiple large cohort studies controlling for cardiovascular disease have repeatedly shown hearing loss to be an independent risk factor for the development of dementia (2–5).

### Neuroanatomic Basis for Sensory and Cognitive Impairments

The neuroanatomy of sensory processing provides context for why multiple sensory impairments occur concomitantly in the context of cognitive impairment. Bilateral vestibular loss is linked to hippocampal atrophy detectable on MRI (37, 38). Olfaction and neurocognition are also linked anatomically and pathophysiologically, particularly concerning Alzheimer's disease and related dementias (39–41). Abnormal amyloid and tau proteins deposit in the olfactory bulb and tract prior to cognitive decline (42). As regional involvement increases, so does the extent of cognitive decline. As the disease progresses, additional neurofibrillary tangles develop in the entorhinal cortex and hippocampal-related structures (43). These are the same anatomic areas through which olfaction is processed. Olfactory identification deficits correlate with atrophy of the hippocampus, olfactory bulb, and entorhinal cortex (44, 45). Behavioral and functional data both indicate that activation of the primary olfactory cortex depends on attention (46, 47). The anterior cingulate cortex, which is activated when cognitive demand is high (48), and during working memory tasks is also activated by olfactory stimuli (49). The increased workload of maintaining appropriate attention and memory as neurocognitive decline progresses may occur at the expense of specific aspects of olfactory and other sensory performance (50). In the context of hearing loss, loss of high-SR auditory fibers, as occurs in age-related hearing loss, is implicated in the development of imbalances in excitation and inhibition in ascending central pathways. This imbalance may lead to a decrease in central gain, dysregulation of the hypothalamic-pituitary axis, decrease in hippocampal long-term potentiation, and an overall decrease in signal-noise ratio (51).

### Contextualizing With Prior Epidemiologic Research

Prior studies have investigated multiple sensory domains including hearing, touch, olfaction, vision, and even taste (52), and found that subjects with multiple sensory impairments have worse neurocognitive outcomes. Our data are consistent in demonstrating the individual associations of hearing, balance, and olfaction with cognitive impairment. Additionally, the highest risk of neurocognitive impairment was in subjects with deficits in all three of the sensory domains tested. This suggests that the effects of multiple sensory impairments may be additive toward odds of CI.

The Epidemiology of Hearing Loss Study (EHLS) demonstrated that olfactory dysfunction predicted the development of cognitive impairment in a cohort of patients prospectively followed for a 5-year period (2). In addition to cognitive impairment, studies of community-dwelling elders have also shown increased morbidity and mortality in subjects with olfactory dysfunction (53–56).

Vestibular dysfunction has been shown to correlate strongly with CI and dementia (9, 57–59). The vestibular system has been independently studied in the context of aging and cognition. Dysfunction is associated with an increased risk of cognitive impairment, dementia, and Alzheimer's disease. Vestibular loss–particularly impairment of the saccule–also predicts poorer spatial cognition in a subset of patients with Alzheimer's disease (57, 58). Cross-sectional analysis of 3 prospective cohort studies on aging populations demonstrated a link between vestibular decline and cognitive decline: the Baltimore Longitudinal Study on Aging (BLSA) (6), the National Health Interview Survey (8), and National Health and Nutrition Examination Survey (60). Within a cross-sectional analysis of the BLSA, an association between olfaction and motor function was identified (61).

#### A Link Between Olfactory Impairment and Cognitive Decline

Subjects with baseline olfactory impairment are more likely to develop cognitive impairment during longitudinal follow-up. Pooled analysis of 8 cohort studies (2, 62–68) encompassing 13,165 participants demonstrated a relative risk of 2.37 (95% CI = 1.91–2.94) for accelerated cognitive decline when subjects with olfactory impairment were followed longitudinally (69). Odor detection is associated with word recall and orientation scores on the Alzheimer's Disease Assessment Scale (70). Odor detection and identification is also correlated with blood flow to the left temporal lobe, entorhinal cortex, and frontal lobes; and activation of the right anterior piriform cortex on fMRI.

### Gait, Balance, and a Testing Proxy for Vestibular Function

“Vestibular Cognition” as a concept is evolving (71) to encompass the peripheral end organs as well as projections through the brainstem to a widespread distribution in higher cortical centers. Low-level reflexes such as the vestibulo-ocular reflex (72) and vestibulospinal reflex (73) stabilize gaze and posture, respectively. These reflexes, along with proprioceptive and visual input interface with higher-order projections to the cerebellum and somatosensory cortex to provide a “sense” of balance. This complex interplay between lower brainstem reflexes and higher-order cortical processing makes contextualizing balance and cognition challenging. For this reason, the Timed-Up-and-Go test was felt to be a simple, easily interpretable proxy for gait and balance function. Due to the need for visual, proprioceptive, and vestibular coordination, this single test was utilized in the present study (13). No studies to date have looked at balance in the context of multisensory impairment, and none of the previously mentioned prospective cohorts have included balance and vestibular dysfunction alongside hearing and olfaction during analyses. Our results show that individual sensory deficits are significantly correlated to co-incident cognitive impairment. More interesting, the effects of multiple sensory impairments appear to be additive in the odds of having CI.

### The Potential for Sensory Rehabilitation

Hearing, olfactory, and vestibular impairments are critically important to recognize due to their potentially modifiable nature. Each of the sensory impairments represents a unique opportunity to intervene and improve outcomes. Our data demonstrate an OR for cognitive impairment of 15.7 in subjects with deficits in three sensory modalities vs. an OR of 5.32 for those with deficits in only two. This indicates that referring subjects with multisensory impairment for hearing restoration *via* hearing aids, vestibular therapy, and/or olfactory training may meaningfully modify the risk of neurocognitive decline. Hearing aids have been shown to potentially mitigate the risk for cognitive decline (74–76). Mertens et al. (77) showed that cochlear implantation in cognitively impaired patients could slow, and even reverse, cognitive changes associated with aging. Olfactory training has been shown to improve olfaction and increase neural connectivity within and between brain regions. When comparing performance on assessments of cognition, depression, overall brain health, and olfaction of non-cognitively impaired community-dwelling senior citizens, 6 months of olfactory training was superior to 6 months of sudokus (78). Olfactory stimulation with scent-impregnated patches placed on the sternum has also been shown to improve vestibular performance and decrease fall risk (79). Taken together, this evidence further highlights the importance of assessing and rehabilitating multisensory dysfunction.

### Limitations of the Present Study

This study is not without limitations. Due to its cross-sectional nature, we are unable to comment on the long-term outcomes of the included subjects. There is an inherent risk of bias during subject recruitment as not all eligible subjects were willing to participate in the study. A degree of selection bias is present due to the exclusion of a large portion of eligible subjects; approximately 31% of initially screened subjects were included in the final cohort. Olfactory assessment utilized AROMA, a relatively novel objective test of olfactory performance, which could be viewed as a limitation. However, prior studies using AROMA have demonstrated high test-retest reliability and a significant correlation of AROMA results with more commonly used tests like the UPSIT (11, 12). Additionally, the study was underpowered to stratify hearing contribution by severity (Normal, Mild, Moderate, Moderately Severe, Severe, Profound), due to a low number of patients with hearing loss worse than “mild.” Recent literature has suggested that central, rather than auditory processing may be more strongly tied to cognitive status (80), rather than PTA as measured here. Secondary analysis of the Adult Changes in Thought (ACT) study (81) showed that decreased performance status on two dichotic central auditory processing tests predicted a higher likelihood of dementia and Alzheimer's disease.

#### Choice of Cognitive Screening Protocol

To assess cognitive status, the MoCA was utilized. The MoCA is commonly deployed in a screening capacity and is not capable of differentiating between etiologies of cognitive impairment. While a more robust neuropsychological battery would be preferable, adding a 1–2-h evaluation to each subject's clinical visit with the psychometricians needed to perform them was not logistically possible. The MoCA is supported in the literature as superior to other common tests like the Mini-Mental Status Exam because it includes measures of executive function (14). Educational status is known to confound MoCA performance with higher education levels positively correlated with MoCA score. Higher education status was found to be a contributing protective factor for the identification of CI in our study, with an OR of 0.22, 95% CI [0.07, 0.58], *p* < 0.001, and was controlled for during multivariate modeling. Future studies will include longitudinal data as well as the impact of sensory deficit-specific rehabilitation of cognitive outcomes. Patients with severe or profound hearing loss were planned to undergo the modified MoCA for the hearing impaired (MoCA-HI) that has previously been validated in this population (82). However, there were no patients with hearing levels worse than “Moderately Severe” recruited, when using the best hearing ear for classification purposes. On retrospective review of the study's recruitment practices, patients with more severe hearing were more commonly funneled into visits for cochlear implant evaluations and so were not as easily captured during enrollment. These subjects are now recruited in a more targeted fashion and are the topic of future study.

## Conclusions

Multisensory impairment is common and associated with cognitive impairment. Deficits in hearing, balance, and olfaction significantly increase the odds of co-incident cognitive impairment vs. those with deficits in fewer domains. When considering single sensory deficits, olfactory dysfunction was the strongest predictor of cognitive impairment. The significance of these findings is in their immediate clinical applicability. These sensory impairments are testable in a point of care fashion and are amenable to rehabilitation. Assessment of multisensory impairment in patients presenting with subjective loss in any of these domains should be considered to facilitate subsequent therapeutic intervention to improve sensory impairments and potentially prevent cognitive decline.

## Data Availability Statement

The raw data supporting the conclusions of this article will be made available by the authors, without undue reservation.

## Ethics Statement

The studies involving human participants were reviewed and approved by Institutional Review Board (IRB), University of Kansas Medical Center. The patients/participants provided their written informed consent to participate in this study.

## Author Contributions

JL conceived the study and coordinated study design, performed statistical analysis, wrote, and critically revised the manuscript. ZA designed data acquisition protocols and critically revised the manuscript. AA and KY critically revised the manuscript. NF performed statistical analysis and critically revised the manuscript. LD'S, HS, and JV conceived the study, participated in study design and data acquisition protocols, and critically revised the manuscript. JV conceived the study, participated in study design and data acquisition protocols, performed statistical analysis, wrote, and critically revised the manuscript. All authors contributed to the article and approved the submitted version.

## Funding

Fees for open access publication are provided at the expense of the Department of Otolaryngology-Head and Neck Surgery at the University of Kansas Medical Center.

## Conflict of Interest

JV discloses intellectual property and a filed patent (17/281121–“Olfactory Diagnostic and Training Kits and Methods”) related to the objective olfactory testing methods used in this research. The remaining authors declare that the research was conducted in the absence of any commercial or financial relationships that could be construed as a potential conflict of interest.

## Publisher's Note

All claims expressed in this article are solely those of the authors and do not necessarily represent those of their affiliated organizations, or those of the publisher, the editors and the reviewers. Any product that may be evaluated in this article, or claim that may be made by its manufacturer, is not guaranteed or endorsed by the publisher.
